# Brain-derived neurotrophic factor (BDNF) has direct anti-inflammatory effects on microglia

**DOI:** 10.3389/fncel.2023.1188672

**Published:** 2023-06-19

**Authors:** Tryston Charlton, Natalie Prowse, Ashley McFee, Noora Heiratifar, Teresa Fortin, Carley Paquette, Shawn Hayley

**Affiliations:** Department of Neuroscience, Carleton University, Ottawa, ON, Canada

**Keywords:** microglia, BDNF, cytokine, primary culture, neurodegeneration, neuroimmune, LPS

## Abstract

Microglia are the primary immunocompetent cells that protect the brain from environmental stressors, but can also be driven to release pro-inflammatory cytokines and induce a cytotoxic environment. Brain-derived neurotrophic factor (BDNF) is important for the regulation of plasticity, synapse formation, and general neuronal health. Yet, little is known about how BDNF impacts microglial activity. We hypothesized that BDNF would have a direct modulatory effect on primary cortical (Postnatal Day 1-3: P1-3) microglia and (Embryonic Day 16: E16) neuronal cultures in the context of a bacterial endotoxin. To this end, we found that a BDNF treatment following LPS-induced inflammation had a marked anti-inflammatory effect, reversing the release of both IL-6 and TNF-α in cortical primary microglia. This modulatory effect was transferrable to cortical primary neurons, such that LPS-activated microglial media was able produce an inflammatory effect when added to a separate neuronal culture, and again, BDNF priming attenuated this effect. BDNF also reversed the overall cytotoxic impact of LPS exposure in microglia. We speculate that BDNF can directly play a role in regulating microglia state and hence, influence microglia-neuron interactions.

## Introduction

Immunity within the central nervous system (CNS) is dependent upon the brain’s specialized microglial cells, which rapidly respond to perturbations in their microenvironment (Zarruk et al., 2018; Dwyer et al., 2020) and have a diverse range of pro-inflammatory and anti-inflammatory states (Saitgareeva et al., 2020). In addition to their role as immune defenders of the CNS, microglia also have critical roles in synaptic plasticity, neurogenesis, as well as neural circuit maintenance and function (Schafer et al., 2012; Diaz-Aparicio et al., 2020). Accordingly, excessive microglia activation and release of inflammatory cytokines has repeatedly been linked to neurological and neuropsychiatric illnesses, such as depression; but unfortunately a causal link is often lacking in many studies (Streit et al., 2004; Hayley and Anisman, 2005; Franklin et al., 2018; Serafini et al., 2020; Hayley and Sun, 2021).

Brain-derived neurotrophic factor normally binds to the receptor tropomyosin receptor kinase B (TrkB) which is found on neurons (Brigadski and Leßmann, 2020); however, there is also evidence that TrkB expression occurs on microglia (Gomes et al., 2013; Prowse and Hayley, 2021). In fact, recent research has found that disruption of the TrkB pathway in microglia affects their activation state and production of pro-inflammatory cytokines (Wu et al., 2020). However some earlier work oppose these findings (Frisén et al., 1993), and there is still a great deal not known of the role of BDNF in microglia. That said although sparse, some recent findings are consistent with microglia expressing TrkB. Specifically, TrkB labeling was found to be co-localized in IBA1+, CD11b+, and OX-42+ rodent brain sections and levels were affected by the age of the animal, as well as being altered in the context of poststroke depression or with exposure to cyclophosphamide (Ding et al., 2020; Wu et al., 2020; Zhu et al., 2020).

A majority of studies done using classical inflammatory stimulators, such as lipopolysaccharide (LPS), have shown an overall decreases in expression of BDNF, but a few have found an upregulation (Gomes et al., 2013; Parkhurst et al., 2013; Hu et al., 2020). Upon encountering an activating stimulus, microglia normally undergo a dramatic change from a ramified morphology to amoeboid and various other intermediates states (Orihuela et al., 2016). A balance between the fluid pro-inflammatory and anti-inflammatory states is required for limiting cell death and preventing irreparable damage to important brain regions (Aloisi, 2001; Block et al., 2007).

Much evidence indicates that BDNF can act directly on neurons to convey neuroprotective consequences. Yet, very little is known about how BDNF can impact microglial cells and how this in turn, might influence neurons. Hence, we sought to assess whether some of the beneficial effects of BDNF might be related to microglial activation state. Specifically, can BDNF directly prime microglial cells to adopt a protective and/or anti-inflammatory phenotype and is this transferrable to neurons. This involved a unique cell culture method, wherein the impact of LPS and BDNF upon cytokine levels was first directly assessed in primary cortical microglial cells. Secondly, the extracellular microglial media was transferred to primary cortical neurons to then directly assess the impact of primed microglial soluble factors on neurons.

## Materials and methods

### Animals and primary culture

C57BL/6 mice were purchased from Charles River Laboratories and were used for *in vitro* culture. Animals were left on a normal 12-h light cycle, given ab libitum access to water and lab chow. Animals were harem mated for 2–14 days for neuronal and microglial cultures. Cortical neuron tissue was collected from dams that were euthanized by rapid decapitation and 5–10 embryonic Day 16 (E16) pups retrieved (per litter). Microglial cultures were collected from 5 to 10 postnatal Day 1-3 (P1-3) pups (per litter).

Primary cultures were produced in 24 well plates on autoclaved, dH_2_O rinsed, coverslips. Both E16 primary neurons and P1-3 microglia were seeded at a concentration of 1 × 10^5^ cells/ml for final assays. Each 6 well row on a 24 well plate was assigned either vehicle, BDNF, LPS or LPS plus BDNF treatments.

The method of collection of cortical mouse neurons was adapted from Hilgenberg and Smith (2007) as well as Gaven et al. (2014), with primary neurons being isolated from the cortex, mildly trypsinized with trypLE and seeded in Poly-D-lysine (PDL) coated wells (for 96-well plates) or acid-etched, PDL-coated glass coverslips (for 24 well plates) at 10 μg/ml in complete neurobasal media (CNB: 2% B-27 [Gibco], 0.5% penicillin/streptomycin, 0.25% GlutaMAX, 97.25% Neurobasal media). E16 cortical neurons were incubated for 7–10 days (DIV7-10) in a humidified environment (37*^o^*C, 5% CO_2_). New CNB, without GlutaMAX, was placed on the cells the day after surgery to remove debris and then every 2–3 days and on DIV7-10 cells were utilized for experiments (Hilgenberg and Smith, 2007; Gaven et al., 2014).

Collection methods for primary P1-3 microglia was adapted from Saura et al. (2003) and Schildge et al. (2013). Briefly, mixed glia were harvested from P1 pups and seeded in T75 flasks containing dissociated cells from 1 to 3 cortices in complete media (10% fetal bovine serum [FBS], 1% penicillin/streptomycin, 89% high glucose DMEM [Gibco]) with full media change after DIV1 and 1/2 media changes every 4–6 days. At 21 DIV, microglia were harvested via mild trypsinization (trypsinization solution: 1:1:1 dilution ratio of DMEM, versene EDTA, and 0.25% trypsin) to yield highest confluency of microglia (Saura et al., 2003). The microglia were then plated immediately into PDL-coated well plates at 1.0 × 10^5^ cells/ml for experimental use in a serum-free microglia media which renders a more *in vivo*-like morphology (Saura et al., 2003; Schildge et al., 2013).

### Treatment conditions

Neurons and microglia were cultured separately in 24 well plates, before microglial media was either assayed or transferred to neurons. Microglia cultures were first treated for 24 h with either (1) vehicle (PBS) or (2) LPS at 100 ng/ml. Thereafter, the media was changed and microglial cells were further treated with either (3) BDNF at 50 ng/ml, or (4) vehicle for another 24 h. Half of the microglia cultures were then assessed for cytokine and LDH levels, while the microglial media from the remaining cultures was transferred to separate neuronal cultures for a final 24 h. Hence, neurons were treated for 24 h with media from either: (1) vehicle treated microglial media, (2) media from microglia primed with LPS at 100 ng/ml, (3) media from microglia primed with BDNF at 50 ng/ml, or (4) media from microglia that were pre-treated with LPS (100 ng/ml) followed by secondary treatment with BDNF (50 ng/ml).

### Measurement of cytokines by ProQuantum immunoassay

Levels of TNFα, IL-6, and IL-4 in the media after treatment were measured using ProQuantum immunoassay kits (Thermo-Fischer). Media was collected from each experimental group and control. Levels of TNFα, IL-6 and IL-4 were measured according to manufacturer’s instruction.

### Lactate dehydrogenase (LDH) assay

Cell activation and toxicity within microglia culture was quantified using the LDH assay (Thermo-Fischer). To measure LDH levels, microglia were seeded in triplicate on a 96 well plate at 5 × 10^4^ cells/ml. After treatments, 50 μl of media was collected from each group and added to a 96-well plate, followed by 50 μl of substrate media and incubated for 30 min at room temperature in the dark. At the end of incubation, stop-buffer was added, and the plate was read at 490 and 680 nm. Percentage of cytotoxicity was calculated by subtracting background absorbance (680 nm) from the absorbance of the medium from cell treatment (490 nm) and dividing by the maximum LDH release which accounts for spontaneous release of LDH from untreated microglia. Maximum LDH release was obtained by adding lysis buffer (1:10) to treatment groups 45 min prior to end of experiment.

### Statistical analysis

All data was analyzed by two-way ANOVA: (Vehicle vs. LPS) vs. (Vehicle vs. BDNF), with significant interactions further analyzed by means of Fisher’s LSD planned follow up comparisons (*p* < 0.05) where appropriate. All data was analyzed using the statistical software Prism (version 9), all data is presented in the form of marginal mean ± standard error of the mean (mean ± SEM), and differences were considered statistically significant when *p* < 0.05.

## Results

**FIGURE 1:**
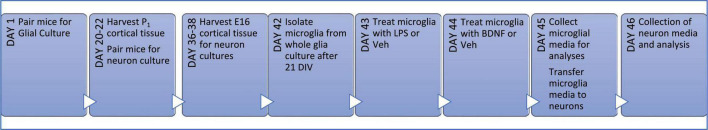
Primary culture timeline. Wild type (WT) littermates were harem-mated for up to 1 week to ensure pregnancy and then cortical tissue was extracted from the pups on day of birth (P1) for whole glial cultures. WT littermates for neuron cultures were harem-mated on the day of glia harvest for 2 days to ensure the day of pregnancy is known; on the 16th day of pregnancy (E16) embryos were collected and neuronal cultures seeded. After 21 days of proliferation (21 DIV), microglia were isolated from whole glial culture into serum-free media and the following day they were treated with LPS or vehicle (Veh) for 24 h; followed by a media change and then either BDNF or Veh treatment for a further 24 h. Microglial media was then transferred to neurons cultures for a final 24 h before all media was collected.

The analysis revealed a significant LPS × BDNF interaction for IL-6 levels in primary microglia (*F*(1,8), = 112.3, *p* < 0.01). Indeed, as shown in Figure 1 microglial levels of IL-6 were remarkably increased in the LPS treated microglial cells, relative to controls (*p* < 0.05). Most importantly, this effect was completely prevented by the subsequent BDNF treatment in the LPS primed microglia. A significant LPS × BDNF interaction was also evident for IL-6 levels within primary neurons (*F*(1,8), = 26.48, *p* < 0.01). The neuronal levels of IL-6 were significantly elevated (albeit at much lower overall concentrations) following exposure to LPS primed microglial media (*p* < 0.05). However, exposure of neurons to media from LPS primed microglia that also received BDNF failed to elicit any IL-6 change from vehicle exposure.

**FIGURE 2:**
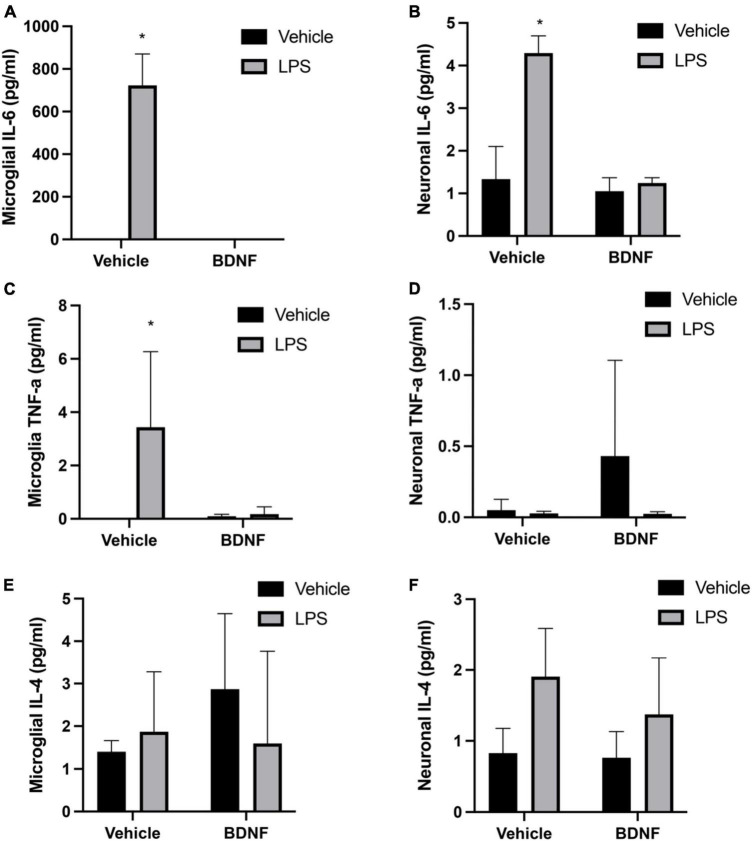
Lipopolysaccharide (LPS) and BDNF influence cytokine levels in primary microglia and neurons. The LPS treatment increased microglial levels of IL-6 and TNF-α, but subsequent BDNF exposure reversed this effect (**A,C**, respectively). No differences were evident for microglial IL-4 **(E)**. Transfer of LPS (100 ng/ml) stimulated microglial media to separate neuronal cultures increased IL-6 levels, but transfer of LPS (100 ng/ml) + BDNF (50 ng/ml) microglial media had no such effect **(B)**. No significant group differences were evident for neuronal TNF-α levels **(D)**. Surprisingly, transfer of LPS primed microglial media to neurons did cause a small but significant rise in IL-4 levels, with no effect of BDNF **(F)**. Vehicle treatment was PBS. *n* = 3 separate cultures/group. *p < 0.05, relative to vehicle treatment.

The interaction between the LPS and BDNF treatments just missed statistical significance for TNF-α levels within primary microglia (*F*(1,8) = 4.13, *p* = 0.07). Given our *a priori* hypothesis and the fact that LPS treatment alone did provoke an obvious TNF-α elevation in microglia (albeit variable), we conducted *post hoc* comparisons. This revealed that LPS treatment increased TNF-α levels in microglia culture compared to vehicle treatment (*p* < 0.05; Figure 2) and once again, this was completely reversed by BDNF exposure. In the case of the cultured neurons, there was no significant differences in TNF-α levels evident in response to the microglial media treatments added.

The ANOVAs failed to reveal any significant interaction between the groups for IL-4 levels within the microglial cultures. However, there was a modest but significant main effect for LPS treatment in the neuronal cultures (*F*(1,8) = 6.26, *p* < 0.05). Indeed, treatment of the neuronal cultures with LPS primed microglial media modestly elevated neuronal IL-4 levels above that of vehicle exposure (*p* < 0.05) (Figure 2).

**FIGURE 3:**
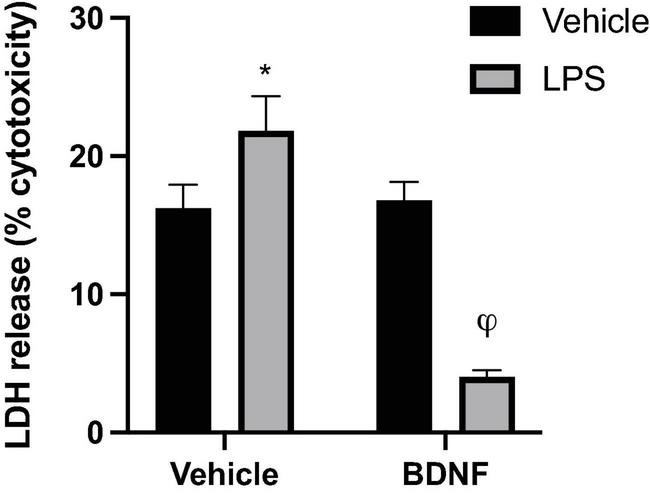
Lipopolysaccharide and BDNF influenced LDH release in primary microglia. Release of the cytoplasmic enzyme, lactate dehydrogenase (LDH), into the microglia media indicated that LPS (100 ng/ml) treatment induced some membrane leakiness and potential cytotoxicity. In contrast, when the LPS-treated cells were also exposed to BDNF this effect was not only reversed, but LDH release was diminished compared to all vehicle treated cells. *n* = 3 separate cultures/group. Vehicle treatment was PBS. *p < 0.05, relative to vehicle only treatment, ^φ^*p* < 0.01, relative to all other groups.

Analysis of LDH levels in the media was used as an index of microglial membrane leakiness and energetic activity, which is linked to potential cytotoxicity. A significant LPS × BDNF interaction was found for LDH levels in the microglial culture (*F*_1_,_8_ = 91.35, *p* < 0.0001). Specifically, LPS exposure significantly increased LDH release from microglia compared to vehicle treated microglia media (*p* < 0.05), but the additional BDNF treatment together with LPS promoted greatly reduced LDH levels, to an extent even below vehicle exposure controls (*p* < 0.05; Figure 3).

## Discussion

Numerous reports demonstrate that BDNF provides trophic support by binding to its TrkB receptor on neurons (Lu et al., 2015; Xu et al., 2017; Jin, 2020). Yet, very little is known regarding the impact of BDNF upon astrocytes or microglia. We presently found that LPS induced the expected robust inflammatory response in cultured primary microglia but most importantly, BDNF treatment completely ameliorated this effect. Indeed, exposure of microglia to BDNF reversed the LPS induced elevations of extracellular released TNF-α and IL-6. A similar effect was observed in primary neurons, wherein an inflammatory response was triggered by exposure to media from LPS activated microglia and this was reversed when media came from LPS + BDNF primed microglia. It should be noted however, that the concentration of IL-6 in microglia media was 100 times higher than that found in the neuronal media. Indeed, neurons appear be able to release very small concentrations of cytokines when sufficiently stressed (Ringheim et al., 1995; Stow et al., 2009). Interestingly, IL-4 levels (while not affected by BDNF) were somewhat increased in the neuronal media after exposure to media from LPS-activated microglia, suggesting the possibility that a protective anti-inflammatory neuronal response was elicited.

Consistent with the present findings, Wu et al. (2020), showed that *in vitro* treatment of “microglia-like” BV2 cells with BDNF attenuated the impact of LPS. Interestingly, they showed that BDNF as a pre-treatment to LPS, rather than post-treatment (as in the current study), produced the greatest reduction in pro-inflammatory cytokine production. This is consistent with BDNF being an important messenger between neurons and glia; particularly during times of infection, injury or cellular stress. For instance, inflammatory-associated damage of neurons causes the release of ATP that can induce the release of BDNF from microglia via purinergic receptors (Fields and Burnstock, 2006; Calovi et al., 2019). The release of trophic factors, such as BDNF, from microglia can then enhance neuronal plasticity to either aid in neuronal survival or compensate for any neuronal loss (Ferrini and De Koninck, 2013; Parkhurst et al., 2013; Kato et al., 2016).

We found that LPS elevated the levels of lactate dehydrogenase (LDH) in microglia media, indicating enhanced release owing to either diminished cell integrity and/or bioenergetic changes in microglia. Microglia can utilize lactate to sustain energy demands and its enzyme, LDH, is involved in catabolizing lactate into pyruvate which is required for the production of oxidative stress factors upon LPS exposure (Monsorno et al., 2022). Indeed, LPS was previously reported to increased LDH activity and enhance its leakage into the extracellular compartment (Takaki et al., 2012; Li et al., 2019). Remarkably however, the BDNF treatment not only reversed this LPS effect, but actually reduced LDH levels below that of controls. This suggests that BDNF may be modulating mechanisms activated by LPS to such a degree that even the basal level of cellular toxicity that is simply associated with the culturing process itself was inhibited. Yet, it should be noted that BDNF alone had no impact of LDH levels, indicating that the impact of the trophic factor was only evident in the context of an LPS provoked inflammatory environment.

To the best of our knowledge, few studies have directly assessed whether BDNF can modulate the activation state of microglia (Trang et al., 2012; Long et al., 2020). As far as we are aware, our study also presents the previously unknown finding that media from LPS and BDNF activated microglia can directly impact neuronal cytokine production. Our findings suggest that BDNF priming of microglia may reprogram inflammatory state and consequently alter neuron-microglia communication. Since our study is a brief communication, we acknowledge that it has limitations, such as our limited focus on cytokines that precludes knowing what other immune factors and possible neurotoxic mediators might have been affected by the LPS and BDNF treatments. It is also unclear as to exactly which elements in the microglial derived media that might drive inflammatory processes at the level of the neuron and what sort of neuropathology might eventually ensue. That said, it seems that BDNF can directly regulate microglial cytokine responses and that this might secondarily impact neurons.

## Data availability statement

The raw data supporting the conclusions of this article will be made available by the authors, without undue reservation.

## Ethics statement

The use of animals in this study was reviewed and approved by the Carleton University Animal Care Committee in accordance with the Canadian Council on Animal Care.

## Author contributions

SH and TC wrote and edited the manuscript. TC, AM, and NH conducted the experimental assays. NP conducted the validation assays. CP, NP, and TF provided the technical assistance. SH conceived and supported the experiments funds. All authors contributed to the article and approved the submitted version.

## References

[B1] Aloisi F. (2001). Immune function of microglia. *Glia* 36 165–179. 10.1002/glia.1106 11596125

[B2] Block M. L. Zecca L. Hong J. S. (2007). Microglia-mediated neurotoxicity: Uncovering the molecular mechanisms. *Nat. Rev. Neurosci.* 8 57–69. 10.1038/nrn2038 17180163

[B3] Brigadski T. Leßmann V. (2020). The physiology of regulated BDNF release. *Cell Tissue Res.* 382 15–45. 10.1007/S00441-020-03253-2 32944867 PMC7529619

[B4] Calovi S. Mut-Arbona P. Sperlágh B. (2019). Microglia and the purinergic signaling system. *Neuroscience* 405 137–147. 10.1016/J.NEUROSCIENCE.2018.12.021 30582977

[B5] Diaz-Aparicio I. Paris I. Sierra-Torre V. Plaza-Zabala A. Rodríguez-Iglesias N. Márquez-Ropero M. (2020). Microglia actively remodel adult hippocampal neurogenesis through the phagocytosis secretome. *J. Neurosci.* 40 1453–1482. 10.1523/JNEUROSCI.0993-19.2019 31896673 PMC7044727

[B6] Ding H. Chen J. Su M. Lin Z. Zhan H. Yang F. (2020). BDNF promotes activation of astrocytes and microglia contributing to neuroinflammation and mechanical allodynia in cyclophosphamide-induced cystitis. *J. Neuroinflamm.* 17:19. 10.1186/s12974-020-1704-0 31931832 PMC6958761

[B7] Dwyer Z. Rudyk C. Beauchamp S. Dinesh A. Sun L. Schlossmacher H. (2020). Microglia depletion prior to lipopolysaccharide and paraquat treatment differentially modulates behavioral and neuronal outcomes in wild type and G2019S LRRK2 knock-in mice. *Brain Behav. Immunity Health* 5:100079. 10.1016/J.BBIH.2020.100079 34589856 PMC8474533

[B8] Ferrini F. De Koninck Y. (2013). Microglia control neuronal network excitability via BDNF signalling. *Neural Plast.* 2013:429815. 10.1155/2013/429815 24089642 PMC3780625

[B9] Fields R. D. Burnstock G. (2006). Purinergic signalling in neuron-glia interactions. *Nat. Rev. Neurosci.* 7 423–436. 10.1038/NRN1928 16715052 PMC2062484

[B10] Franklin T. C. Xu C. Duman R. S. (2018). Depression and sterile inflammation: Essential role of danger associated molecular patterns. *Brain Behav. Immunity* 72 2–13. 10.1016/j.bbi.2017.10.025 29102801

[B11] Frisén J. Verge V. M. K. Fried K. Risling M. Persson H. Trotter J. (1993). Characterization of glial trkB receptors: Differential response to injury in the central and peripheral nervous systems. *Proc. Natl. Acad. Sci. U.S.A.* 90 4971–4975. 10.1073/PNAS.90.11.4971 8389459 PMC46635

[B12] Gaven F. Marin P. Claeysen S. (2014). Primary culture of mouse dopaminergic neurons. *J. Visual. Exp.* 91:51751. 10.3791/51751 25226064 PMC4828056

[B13] Gomes C. Ferreira R. George J. Sanches R. Rodrigues D. I. Gonçalves N. (2013). Activation of microglial cells triggers a release of brain-derived neurotrophic factor (BDNF) inducing their proliferation in an adenosine A2A receptor-dependent manner: A2A receptor blockade prevents BDNF release and proliferation of microglia. *J. Neuroinflamm.* 10 1–13. 10.1186/1742-2094-10-16/FIGURES/8 PMC3567964 23363775

[B14] Hayley S. Anisman H. (2005). Multiple mechanisms of cytokine action in neurodegenerative and psychiatric states: Neurochemical and molecular substrates. *Curr. Pharm. Design* 11 947–962. 10.2174/1381612053381611 15777246

[B15] Hayley S. Sun H. (2021). Neuroimmune multi-hit perspective of coronaviral infection. *J. Neuroinflamm.* 18:231. 10.1186/s12974-021-02282-0 34645457 PMC8512650

[B16] Hilgenberg L. G. W. Smith M. A. (2007). Preparation of dissociated mouse cortical neuron cultures. *JoVE* 10:e562. 10.3791/562 18989405 PMC2557074

[B17] Hu Y. Mai W. Chen L. Cao K. Zhang B. Zhang Z. (2020). mTOR-mediated metabolic reprogramming shapes distinct microglia functions in response to lipopolysaccharide and ATP. *Glia* 68 1031–1045. 10.1002/GLIA.23760 31793691

[B18] Jin W. (2020). Regulation of BDNF-TrkB signaling and potential therapeutic strategies for Parkinson’s disease. *J. Clin. Med.* 9:257. 10.3390/JCM9010257 31963575 PMC7019526

[B19] Kato G. Inada H. Wake H. Akiyoshi R. Miyamoto A. Eto K. (2016). Microglial contact prevents excess depolarization and rescues neurons from excitotoxicity. *eNeuro* 3 9133–9144. 10.1523/ENEURO.0004-16.2016 27390772 PMC4916329

[B20] Li C. Zhao B. Lin C. Gong Z. An X. (2019). TREM2 inhibits inflammatory responses in mouse microglia by suppressing the PI3K/NF-κB signaling. *Cell Biol. Int.* 43 360–372. 10.1002/cbin.10975 29663649 PMC7379930

[B21] Long T. He W. Pan Q. Zhang S. Zhang D. Qin G. (2020). Microglia P2X4R-BDNF signalling contributes to central sensitization in a recurrent nitroglycerin-induced chronic migraine model. *J. Headache Pain* 21 1–17. 10.1186/S10194-019-1070-4/FIGURES/9 31937253 PMC6961410

[B22] Lu B. Nagappan G. Lu Y. (2015). BDNF and synaptic plasticity, cognitive function, and dysfunction. *Handb Exp. Pharmacol.* 220 223–250. 10.1007/978-3-642-45106-5_9 24668475

[B23] Monsorno K. Buckinx A. Paolicelli R. C. (2022). Microglial metabolic flexibility: Emerging roles for lactate. *Trends Endocrinol. Metab. TEM* 33 186–195. 10.1016/j.tem.2021.12.001 34996673

[B24] Orihuela R. McPherson C. A. Harry G. J. (2016). Microglial M1/M2 polarization and metabolic states. *Br. J. Pharmacol.* 173 649–665. 10.1111/bph.13139 25800044 PMC4742299

[B25] Parkhurst C. N. Yang G. Ninan I. Savas J. N. Yates J. R. Lafaille J. J. (2013). Microglia promote learning-dependent synapse formation through brain-derived neurotrophic factor. *Cell* 155 1596–1609. 10.1016/j.cell.2013.11.030 24360280 PMC4033691

[B26] Prowse N. Hayley S. (2021). Microglia and BDNF at the crossroads of stressor related disorders: Towards a unique trophic phenotype. *Neurosci. Biobehav. Rev.* 131 135–163. 10.1016/J.NEUBIOREV.2021.09.018 34537262

[B27] Ringheim G. E. Burgher K. L. Heroux J. A. (1995). Interleukin-6 mRNA expression by cortical neurons in culture: Evidence for neuronal sources of interleukin-6 production in the brain. *J. Neuroimmunol.* 63 113–123. 10.1016/0165-5728(95)00134-4 8550808

[B28] Saitgareeva A. R. Bulygin K. V. Gareev I. F. Beylerli O. A. Akhmadeeva L. R. (2020). The role of microglia in the development of neurodegeneration. *Neurol. Sci.* 41 3609–3615. 10.1007/s10072-020-04468-5 32458252

[B29] Saura J. Tusell J. M. Serratosa J. (2003). High-yield isolation of murine microglia by mild trypsinization. *Glia* 44 183–189. 10.1002/glia.10274 14603460

[B30] Schafer D. P. Lehrman E. K. Kautzman A. G. Koyama R. Mardinly A. R. Yamasaki R. (2012). Microglia sculpt postnatal neural circuits in an activity and complement-dependent manner. *Neuron* 74 691–705. 10.1016/J.NEURON.2012.03.026/ATTACHMENT/29EF4910-1D22-44CF-B97A-3A9BFC0F0AB0/MMC3.MOV 22632727 PMC3528177

[B31] Schildge S. Bohrer C. Beck K. Schachtrup C. (2013). Isolation and culture of mouse cortical astrocytes. *JoVE* 71:e50079. 10.3791/50079 23380713 PMC3582677

[B32] Serafini G. Parisi V. M. Aguglia A. Amerio A. Sampogna G. Fiorillo A. (2020). A specific inflammatory profile underlying suicide risk? systematic review of the main literature findings. *Int. J. Environ. Res. Public Health* 17:2393. 10.3390/ijerph17072393 32244611 PMC7177217

[B33] Stow J. L. Ching Low P. Offenhäuser C. Sangermani D. (2009). Cytokine secretion in macrophages and other cells: Pathways and mediators. *Immunobiology* 214 601–612. 10.1016/J.IMBIO.2008.11.005 19268389

[B34] Streit W. J. Mrak R. E. Griffin W. S. T. (2004). Microglia and neuroinflammation: A pathological perspective. *J. Neuroinflamm.* 1:14. 10.1186/1742-2094-1-14 15285801 PMC509427

[B35] Takaki J. Fujimori K. Miura M. Suzuki T. Sekino Y. Sato K. (2012). L-glutamate released from activated microglia downregulates astrocytic L-glutamate transporter expression in neuroinflammation: The “collusion” hypothesis for increased extracellular L-glutamate concentration in neuroinflammation. *J. Neuroinflamm.* 9:275. 10.1186/1742-2094-9-275 23259598 PMC3575281

[B36] Trang T. Beggs S. Salter M. W. (2012). Brain-derived neurotrophic factor from microglia: A molecular substrate for neuropathic pain. *Neuron Glia Biol.* 7 99–108. 10.1017/S1740925X12000087 22613083 PMC3748035

[B37] Wu S. Y. Pan B. S. Tsai S. F. Chiang Y. T. Huang B. M. Mo F. E. (2020). BDNF reverses aging-related microglial activation. *J. Neuroinflamm.* 17 1–18. 10.1186/S12974-020-01887-1/FIGURES/7 PMC7362451 32664974

[B38] Xu D. Lian D. Wu J. Liu Y. Zhu M. Sun J. (2017). Brain-derived neurotrophic factor reduces inflammation and hippocampal apoptosis in experimental Streptococcus pneumoniae meningitis. *J. Neuroinflamm.* 14 1–13. 10.1186/S12974-017-0930-6/FIGURES/9 PMC5545027 28778220

[B39] Zarruk J. G. Greenhalgh A. D. David S. (2018). Microglia and macrophages differ in their inflammatory profile after permanent brain ischemia. *Exp. Neurol.* 301(Pt B) 120–132. 10.1016/J.EXPNEUROL.2017.08.011 28843543

[B40] Zhu H.-X. Cheng L.-J. Ou Yang R.-W. Li Y.-Y. Liu J. Dai D. (2020). Reduced amygdala microglial expression of brain-derived neurotrophic factor and tyrosine kinase receptor b (TrkB) in a rat model of poststroke depression. *Med. Sci. Monitor Int. Med. J. Exp. Clin. Res.* 26:e926323. 10.12659/MSM.926323 33206632 PMC7682116

